# Cellular sheddases are induced by Merkel cell polyomavirus small tumour antigen to mediate cell dissociation and invasiveness

**DOI:** 10.1371/journal.ppat.1007276

**Published:** 2018-09-06

**Authors:** Nnenna Nwogu, James R. Boyne, Samuel J. Dobson, Krzysztof Poterlowicz, G. Eric Blair, Andrew Macdonald, Jamel Mankouri, Adrian Whitehouse

**Affiliations:** 1 School of Molecular and Cellular Biology and Astbury Centre for Structural Molecular Biology, Faculty of Biological Sciences, University of Leeds, Leeds, United Kingdom; 2 Centre for Skin Sciences, School of Chemistry and Biosciences, Faculty of Life Sciences, University of Bradford, Bradford, United Kingdom; Fred Hutchinson Cancer Research Center, UNITED STATES

## Abstract

Merkel cell carcinoma (MCC) is an aggressive skin cancer with a high propensity for recurrence and metastasis. Merkel cell polyomavirus (MCPyV) is recognised as the causative factor in the majority of MCC cases. The MCPyV small tumour antigen (ST) is considered to be the main viral transforming factor, however potential mechanisms linking ST expression to the highly metastatic nature of MCC are yet to be fully elucidated. Metastasis is a complex process, with several discrete steps required for the formation of secondary tumour sites. One essential trait that underpins the ability of cancer cells to metastasise is how they interact with adjoining tumour cells and the surrounding extracellular matrix. Here we demonstrate that MCPyV ST expression disrupts the integrity of cell-cell junctions, thereby enhancing cell dissociation and implicate the cellular sheddases, A disintegrin and metalloproteinase (ADAM) 10 and 17 proteins in this process. Inhibition of ADAM 10 and 17 activity reduced MCPyV ST-induced cell dissociation and motility, attributing their function as critical to the MCPyV-induced metastatic processes. Consistent with these data, we confirm that ADAM 10 and 17 are upregulated in MCPyV-positive primary MCC tumours. These novel findings implicate cellular sheddases as key host cell factors contributing to virus-mediated cellular transformation and metastasis. Notably, ADAM protein expression may be a novel biomarker of MCC prognosis and given the current interest in cellular sheddase inhibitors for cancer therapeutics, it highlights ADAM 10 and 17 activity as a novel opportunity for targeted interventions for disseminated MCC.

## Author summary

The majority of cancer-related deaths occur due to metastatic disease. Therefore, understanding the molecular and cellular mechanisms underlying the process of metastasis is essential to developing new therapeutic interventions to improve cancer patient survival. Merkel cell carcinoma (MCC) is an aggressive and highly metastatic cancer. Merkel cell polyomavirus (MCPyV) has been implicated as the causative agent in the majority of MCC cases. The MCPyV small tumour antigen (ST) is believed to function as the major oncoprotein. However, little is known about the mechanisms through which MCPyV ST may be implicated in causing the high rates of metastatic spread observed in MCC tumours. Here we show that specific cellular sheddases, namely A disintegrin and metalloproteinase (ADAM) 10 and 17 protein levels are increased upon MCPyV ST expression. Moreover, we show that MCPyV ST-induced ADAM 10 and 17 are required to breakdown cell-cell junctions resulting in increased cell dissociation, migration and invasion. As such, ADAM protein expression may provide a novel biomarker of MCC prognosis. In addition, linking cellular sheddases to MCPyV-positive MCC metastasis may provide novel therapeutic interventions.

## Introduction

Merkel cell carcinoma (MCC) is a highly aggressive neuroendocrine cancer of the skin [1]. Although rare, the incidence of MCC has increased over the past twenty years in both Europe and the United States of America [2], attributed to advances in reporting, diagnostic improvements and known risk factors. UV light appears to be an important factor in MCC, with a positive correlation between geographic UVB radiation indices and age-adjusted MCC amongst Caucasians [1, 3]. The predominance of MCC in elderly persons also highlights immunosuppression as an important risk factor, supported by disproportionally higher rates of MCC in patients on long-term iatrogenic immunosuppression, in addition to patients with lymphoproliferative disorders and HIV/AIDs [2]. Due to its aggressive nature MCC carries a high risk of local, regional and distant recurrence [4]. As such, the 5-year survival rates range from 60–87% for local disease to 11–20% for metastatic disease [5–7].

The majority of MCC cases, ~80%, are associated with Merkel cell polyomavirus (MCPyV) [8], whilst the remaining cases contain a high degree of single nucleotide polymorphisms consistent with UV-mediated mutations [9, 10]. MCPyV is a common skin commensal causing an asymptomatic infection usually acquired in childhood. Like other polyomaviruses, MCPyV expresses a variety of early spliced variant regulatory proteins required for viral replication and pathogenesis, including the small and large tumour antigens (ST and LT, respectively) [11]. Upon loss of immunosurveillance, the MCPyV genome integrates into the host genome prior to clonal expansion of tumour cells [12, 13]. A further prerequisite for MCPyV-mediated tumourigenesis is the truncation of the LT antigen rendering the virus replication defective [13]. These truncations lead to the loss of functional LT domains associated with virus replication, although all preserve the LXCXE Retinoblastoma (Rb) protein-binding domain, which alters cell cycle progression contributing to increased cell proliferation [14, 15].

Both MCPyV ST and truncated LT antigens are essential for MCC cell survival and proliferation, exemplified by siRNA-mediated depletion of either protein leading to cell cycle arrest and apoptosis [16]. Moreover, genetically engineered mice expressing MCPyV T antigens in the stratified epithelium display signs of neoplastic progression [17]. However, in contrast to the prototype polyomavirus, simian virus 40 (SV40), MCPyV truncated LT forms cannot initiate cellular transformation alone and function in an accessory role by binding host factors which regulate cellular proliferation, such as Rb and Hsc70 [18, 19]. Conversely, MCPyV ST expression is sufficient to transform rodent cells to anchorage- and contact-independent growth and induce serum-free proliferation of human cells [18]. In addition, preterm transgenic mice co-expressing epidermis-tagged MCPyV ST and the cell fate determinant atonal bHLH transcription factor 1 developed widespread cellular aggregates representative of human intraepidermal MCC [20]. Together these observations show that MCPyV ST is the major oncogenic driver of MCC. Several MCPyV ST-mediated mechanisms contribute to MCC development and proliferation. ST expression leads to the hyperphosphorylation of the translation regulatory protein, 4E-BP1, resulting in dysregulation of cap-dependent translation [18] and prevents SCF^Fwb7^-mediated degradation of MCPyV LT and several cellular oncoproteins [21]. It induces centrosome overduplication, aneuploidy, chromosome breakage and the formation of micronuclei by targeting cellular E3 ubiquitin ligases [22]. MCPyV ST also functions as an inhibitor of NF-κB-mediated transcription [23, 24]. Moreover, ST activates gene expression by associating with MYCL and the EP400 histone and chromatin remodelling complex [25], inducing transcriptional changes effecting for example glycolytic metabolic pathways [26].

The poor survival rates of MCC strongly correlate to the high dissemination rates and metastatic nature of MCC [5]. Whether MCPyV T antigens contribute to MCC metastasis is yet to be fully elucidated. Metastasis is a complex process, with several discrete steps required for the formation of secondary tumour sites [27]. These metastatic hallmarks include loss of cell adhesion, gain of cell motility, dissemination via the vasculature, and colonisation of distant sites [28, 29]. Recent quantitative proteomic studies suggest MCPyV ST expression can promote cell motility and migration [30–32] by inducing differential expression of cellular proteins involved in microtubule [30] and actin-associated cytoskeletal organization and dynamics [31], leading to microtubule destabilization and filopodium formation. These results suggest that MCPyV may be associated with the highly metastatic nature of MCC, and is supported by studies showing that engraftment of MCC cell lines into SCID mice results in circulating tumour cells and metastasis formation [33].

One key trait that underpins the ability of cancer cells to become invasive and metastasise is how they interact with the surrounding extracellular matrix (ECM) and adjoining tumour and stromal cells [34, 35]. Cell–cell junctions are sites of intercellular adhesion that maintain the integrity of epithelial tissue and regulate signalling between cells [36]. The expression of cell adhesion molecules is tightly regulated, as dysregulation of cell adhesion between tumour cells and turnover of the surrounding ECM plays a critical role in malignant transformation and the initiation of the metastatic cascade [37]. A key mediator of cell adhesion in epithelial tissues is E-cadherin and its loss can promote invasive and metastatic behaviour in many epithelial tumours [38]. The cytoplasmic domain of E-cadherin binds to members of the catenin family, linking this multiple protein complex to the actin cytoskeleton through alpha-E-catenin. The clustering of cadherin-catenin complexes on adjacent cells leads to localised actin remodelling required for the formation of adheren junctions [39]. Notably, the loss of E-cadherin and associated cell adhesion molecules, results in the suppression or weakening of cell–cell adhesion which is regarded as a crucial step in the epithelial–mesenchymal transition (EMT) [40, 41], a process enabling a cell to acquire a more migratory and invasive mesenchymal phenotype. Loss of E-cadherin and associated cell adhesion molecules in human tumours is caused by multiple factors, including germline mutations, promoter methylation, downregulation of EMT-associated transcriptional repressor proteins and the upregulation of cellular proteinases causing proteolytic cleavage of cell adhesion molecules [42–44].

ADAMs (a disintegrin and metalloproteinases), are a family of zinc-dependent transmembrane proteins implicated in the ectodomain shedding of various membrane-bound proteins [45]. Of the 21 human largely cell-membrane associated ADAMs, 13 have proteolytic sheddase capacities modulating the activity of membrane cytokines and growth factors, their receptors and cell adhesion molecules, including cadherins, selectins and integrins [46]. ADAM sheddase activities have been implicated in several physiological and pathological processes including inflammation, tumour growth and metastatic progression [47], reinforced by upregulation of proteolytic ADAMs in both tumour tissues and cancer cell lines [48–50]. Correlations exist between levels of specific ADAMs and parameters of tumour progression, implying that these sheddases are implicated in the process of cancer development and the dissemination of metastatic tumour cells [51]. ADAMs are now emerging as potential cancer biomarkers for aiding cancer diagnoses and predicting patient outcome [52]. In addition, selective ADAM inhibitors have promising anti-tumourigenic effects in *in vitro* and *in vivo* studies and are progressing into clinical trials [53].

Here we demonstrate that the cellular sheddases, ADAM 10 and 17, are upregulated in a MCPyV ST-dependent manner. Work highlights the essential role of ADAM sheddases in MCPyV ST-mediated disruption of cell adhesion leading to enhanced cell dissociation and motility. This suggests that ADAM protein expression may be a novel biomarker of MCC prognosis and inhibiting ADAM activity may provide a novel opportunity for targeted interventions for disseminated MCC.

## Results

### MCPyV ST expression induces cell dissociation by disrupting cell junctions

**Fig 1:**
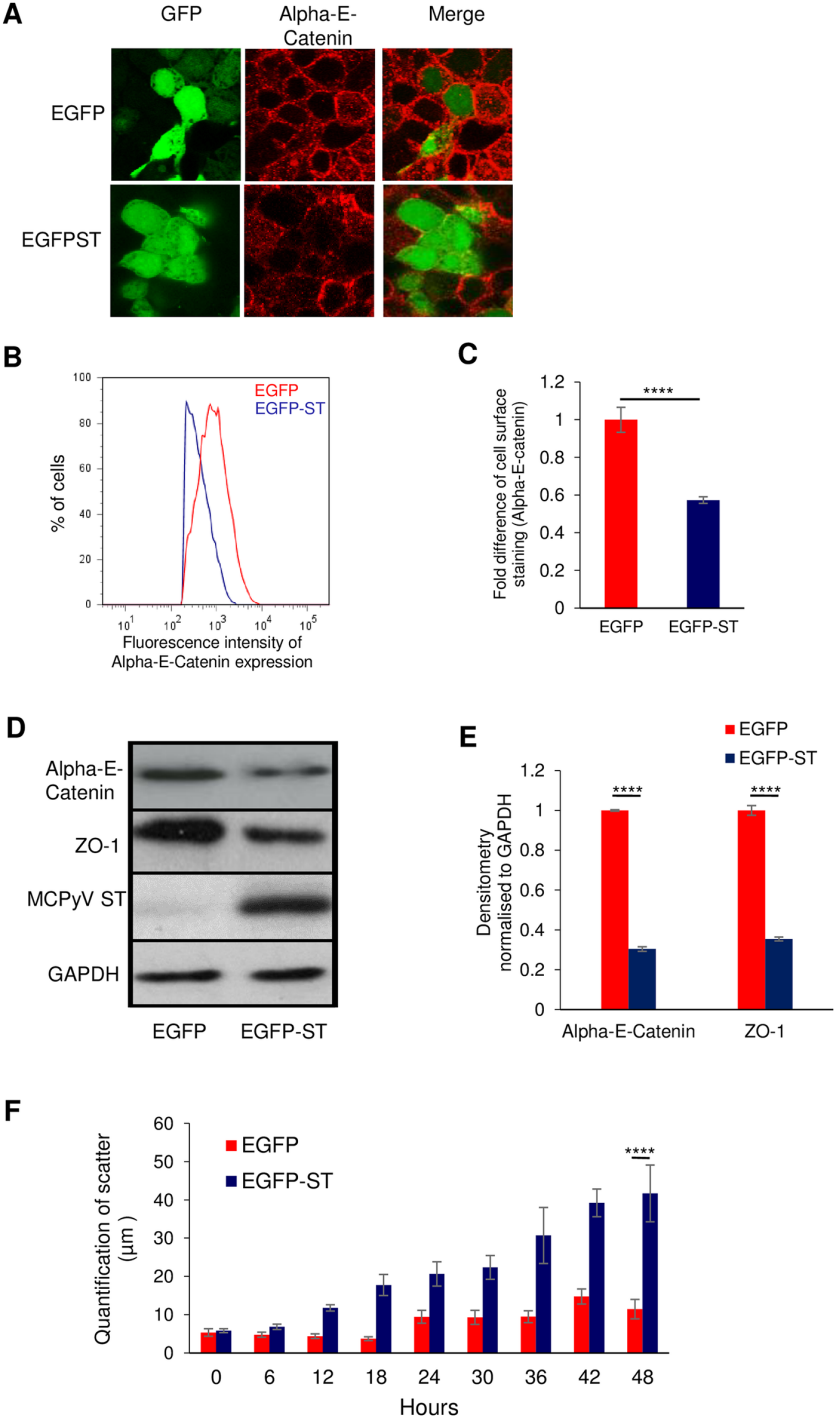
MCPyV ST expression induces cell dissociation by disrupting cell junctions. (A) HEK-293 cells were transfected with 1 μg of pEGFP or pEGFP-ST expression plasmids. 24 h later cells were fixed and GFP fluorescence analysed by direct visualisation, whereas endogenous Alpha-E-catenin was identified by indirect immunofluorescence using a specific antibody. (B) EGFP or EGFP-ST transfected HEK-293 cells were harvested and stained with an Alpha-E-catenin specific antibody and Alexa-Fluor-tagged secondary antibody. Mean fluorescence intensity was analyzed using FlowJo software (C) Fold difference of cell surface staining was calculated using three replicates per experiment, n = 3 by a two-tailed t-test with unequal variance, **** = p≤ 0.0001. (D) HEK 293 cells were transfected with EGFP and EGFP-ST expression plasmids for 48 hours. Immunoblot analysis was performed on the cellular lysates and analysed with Alpha-E-catenin and ZO-1 specific antibodies. GAPDH was used as a measure of equal loading and the 2T2 hybridoma was used to confirm MCPyV ST expression. (E) Densitometry quantification of immunoblots was carried out using the Image J software and is shown as a percentage relative to the loading control, GAPDH. Data analysed using three replicates per experiment, n = 3 and statistical analysis using a two-tailed t-test with unequal variance, **** = p<0.0001. (F) EGFP or EGFP-ST transfected HEK 293 cells were serum starved for 24 hours to induce aggregate formation. Upon reintroduction of serum, cells were fixed and stained with DAPI at 6 hourly intervals. Images were analysed using Image J to quantify the distance between each cell nucleus. Data analysed using three replicates per experiment, n = 50 cells, by a two-tailed t-test with unequal variance, **** = p≤ 0.0001.

Cell-cell adhesion and cell interaction to the extracellular matrix is required for tissue integrity [54]. Disrupting cell-cell adhesion enhances cell scattering, which is essential to initiate cell migration and metastatic spread [55]. To determine whether MCPyV ST expression affects the integrity of cell junctions, EGFP and EGFP-ST transfected HEK 293 cells were stained with an Alpha-E-catenin-specific antibody. Alpha-E-catenin, which is predominantly expressed at the plasma membrane mediating cell adhesion and its breakdown impliess a loss of structural integrity at cell junctions [56]. Results demonstrate that Alpha-E-catenin in control EGFP-expressing cells primarily localised to the plasma membrane, in contrast a reduced and incomplete plasma membrane localisation is observed in EGFP-ST-expressing cells, indicative of diminished cell-cell adhesion (Fig 1A). A similar result was also observed upon inducible MCPyV ST expression in a HEK 293 FlpIn-derived cell line (i293-ST) [30] (S1 Fig). In addition, immunoblotting these cell lysates showed a decrease in Alpha-E-catenin protein levels (S1 Fig). Quantification of Alpha-E-catenin levels at the plasma membrane in EGFP and EGFP-ST-expressing cells was then performed using flow cytometry. Results validated the immunofluorescence data demonstrating a reduction in Alpha-E-catenin levels upon MCPyV ST expression (Fig 1B and 1C). To confirm the disruption of cell junctions, the levels of a second cell adhesion-associated protein, Zona occludin 1 (ZO-1) [57], was compared in EGFP versus EGFP-ST-expressing cells. Consistent with the reduction in Alpha-E-catenin levels, immunoblot analysis showed a significant decrease in ZO-1 expression upon MCPyV ST expression (Fig 1D and 1E). Together, these results provide the first indication that MCPyV ST dysregulates cell-cell adhesion.

Loss of cell junction integrity enhances the ability of a cell to migrate and dissociate from its primary site. To assess whether MCPyV ST induces cell dissociation and scatter, a cell scatter assay was performed as previously described [58]. Here EGFP and EGFP-ST transfected HEK 293 cells were incubated in low serum to induce aggregation, upon reintroduction of serum cells were fixed and stained with DAPI at 6 hourly intervals and clusters of cells were analysed to quantify the distance between each cell nucleus (Fig 1F). Results show that EGFP control cells scarcely dissociate, instead remaining in cell clusters. In contrast, MCPyV ST-expressing cells dissociated significantly from their initial cell clusters. Similar results were also observed in the MCPyV negative cell line MCC13, transfected with either EGFP or EGFP-ST expression constructs (S1 Fig), although results in MCC13 cells were less pronounced than in HEK 293 cells. These results suggest that MCPyV ST expression can lead to the breakdown of cell junctions enhancing cell dissociation.

### MCPyV ST expression affects the levels of ADAM proteins

**Fig 2:**
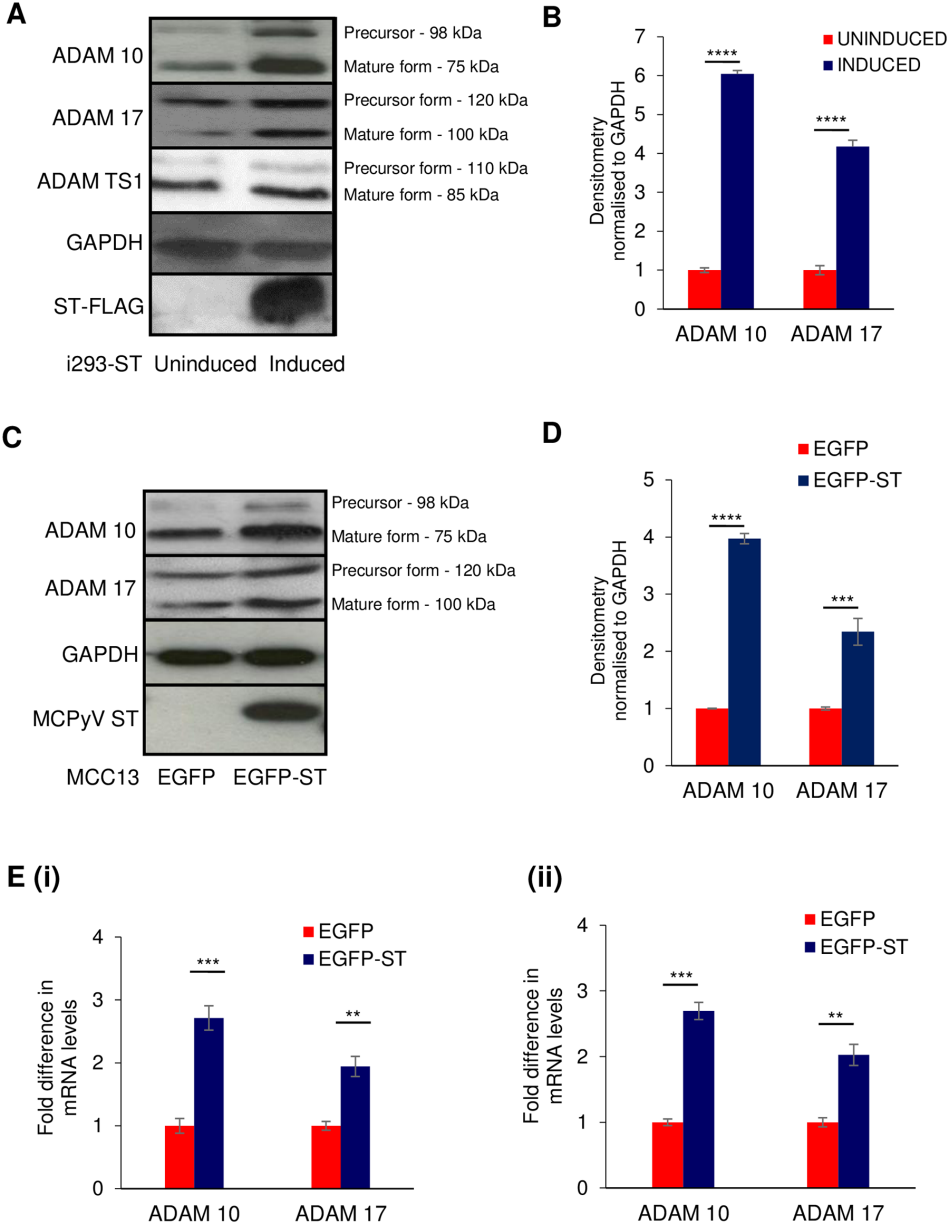
MCPyV ST expression increases the levels of ADAM proteins. (A) i293-ST cells remained uninduced or were incubated for 48 hours in the presence of doxycycline hyclate or (C) MCC13 cells were transfected with 1μg of pEGFP or pEGFP-ST for 12 hours. Cell lysates were then probed with ADAM 10-, ADAM 17- and ADAM TS1-specific antibodies. GAPDH was used as a measure of equal loading, the 2T2 hybridoma was used to confirm MCPyV ST expression. (B and D) Densitometry quantification of immunoblots was carried out using the Image J software and is shown as a percentage relative to the loading control, GAPDH. Data analysed using three replicates per experiment, n = 3 and statistical analysis using a two-tailed t-test with unequal variance, *** = p<0.001, **** = p<0.0001. (E) Total RNA was extracted from EGFP or EGFP-ST transfected (i) HEK 293 and (ii) MCC13 cells and relative transcript levels were analysed by qRT-PCR using GAPDH as a reference. Fold increase was determined by ΔΔCt and statistical significance analysed using a non-paired t-test, *** = p<0.001, ** = p<0.01.

Cellular sheddases function predominantly in the ectodomain cleavage of various membrane-bound proteins, including cell adhesion molecules. Therefore, to identify potential cellular sheddases induced upon MCPyV ST expression, we re-analysed a previously published SILAC-based quantitative proteomic dataset which determined alterations in the host cell proteome upon inducible MCPyV ST expression in a HEK 293 FlpIn-derived cell line (i293-ST) [30]. MCPyV ST expression led to an increase in the levels of two specific cellular sheddases, namely ADAM 10 and 17 proteins by 7.6 and 4.3 fold, respectively (S1 Fig). To confirm an increase in ADAM protein levels upon MCPyV ST expression, cell lysates of uninduced and induced i293-ST cells were analysed by immunoblotting. Results demonstrated a significant increase in ADAM 10 and 17 mature protein levels, compared to ADAM TS1 (Fig 2A). Densitometry-based quantification of the immunoblot analysis showed an increase in the mature forms of ADAM 10 and 17 expression of 6 and 4 fold, respectively (Fig 2B). A similar fold increase was also observed in MCC13 cells, transfected with either EGFP or EGFP-ST expression constructs (Fig 2C and 2D). The increase observed in ADAM protein levels occurs at the transcriptional level, as RT-qPCR showed significant changes in the mRNA levels of both ADAM proteins upon MCPyV ST expression in both HEK 293 and MCC13 cells (Fig 2E), correlating with recent results showing MCPyV ST can dynamically alter the transcriptome of human cells [26].

**Fig 3:**
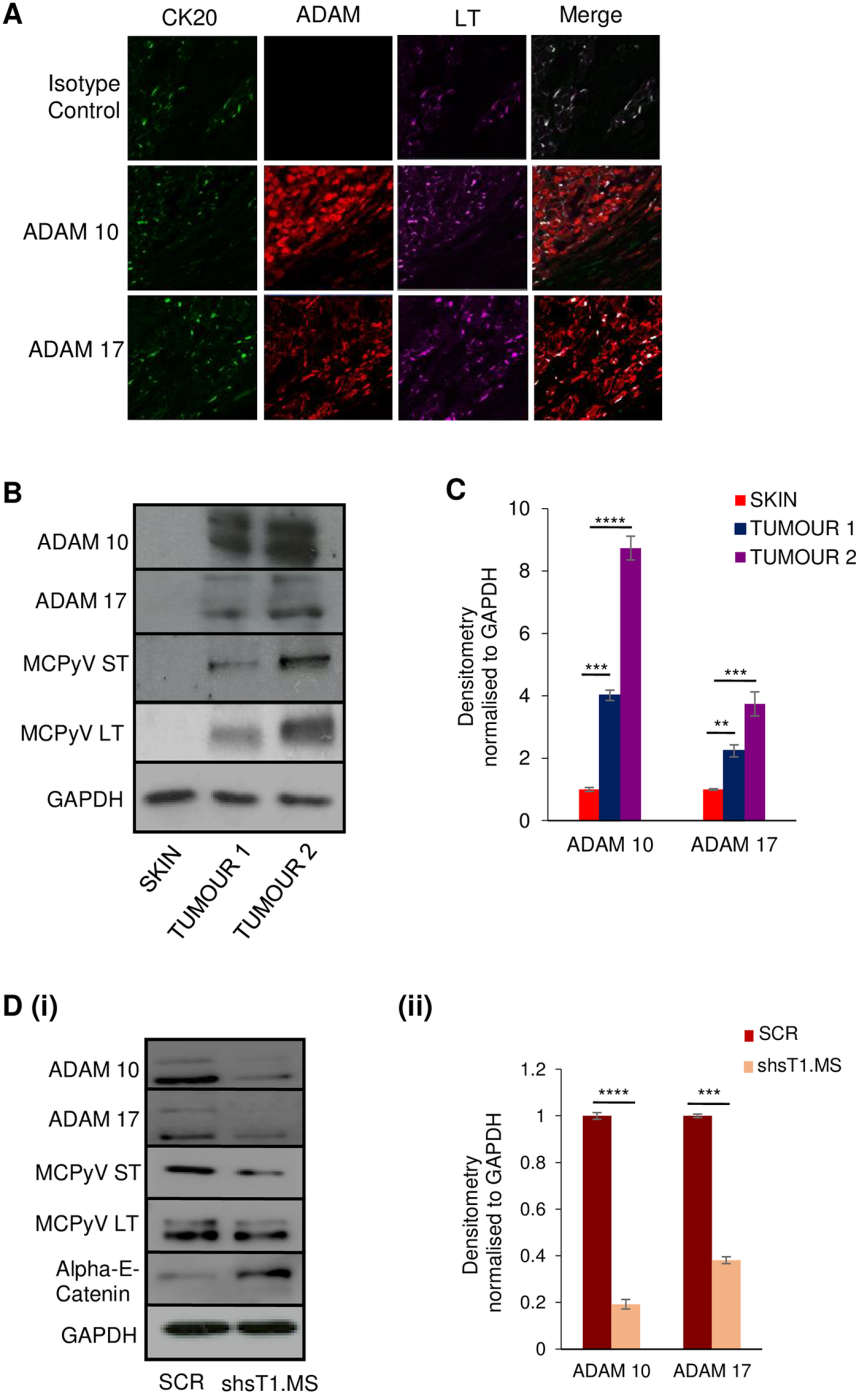
ADAM 10 and 17 levels are dysregulated in MCC tumour samples. (A) FFPE sections of primary MCC tumours were stained with CK20, MCPyV LT and ADAM 10- or ADAM 17-specific antibodies or an isotype negative control. Sections were then incubated with Alexa Fluor labelled secondary antibodies and analysed using a Zeiss LSM880 confocal laser scanning microscope. (B) Immunoblot analysis was performed on the cellular lysates of two independent MCC tumour samples and a negative control non-tumour cadaveric skin sample using ADAM 10- or ADAM 17-specific antibodies. GAPDH was used as a measure of equal loading, the 2T2 hybridoma was used to confirm MCPyV ST expression and the CM2B4 antibody used to confirm MCPyV tLT expression. (C) Densitometry quantification of immunoblots was carried out using the Image J software and is shown as a percentage relative to the loading control, GAPDH. Data analysed using three replicates per experiment, n = 3 and statistical analysis using a two-tailed t-test with unequal variance, **** = p≤ 0.0001, *** = p<0.001, ** = p<0.01. (D) (i) The MCPyV-positive MCC cell line, WAGA, was transduced with lentivirus expressing a scrambled shRNA or ST-targetting shRNA. Upon ST depletion cell lysates were probed with ADAM 10-, ADAM 17- or Alpha-E-catenin specific antibodies. GAPDH was used as a measure of equal loading, the 2T2 hybridoma was used to confirm MCPyV ST expression and the CM2B4 antibody used to confirm MCPyV tLT expression. These samples have been previously used to assess expression of actin-associated proteins [31]. (ii) Densitometry quantification of immunoblots was carried out using the Image J software and is shown as a percentage relative to the loading control, GAPDH. Data analysed using three replicates per experiment, n = 3 and statistical analysis using a two-tailed t-test with unequal variance, **** = p<0.0001, *** = p<0.001.

To further investigate the differential expression of ADAM 10 and 17 proteins in the context of MCC, multicolour immunochemistry analysis was performed on formalin-fixed, paraffin-embedded (FFPE) sections of primary MCC tumours. Sections were stained with ADAM 10 and 17, cytokeratin 20 (CK20) (a marker widely used to distinguish MCC) and MCPyV LT specific antibodies. An isotyped-matched control was also used as a negative control. CK20 staining confirmed MCC status of the sections and results show increased levels of ADAM 10 and 17 expression coincident with LT staining in regions of both MCPyV-positive MCC tumours (Fig 3A). Moreover, immunoblot analysis was performed on cell lysates of two unrelated MCPyV-positive MCC tumour samples comparing protein levels against a negative control non-tumour cadaveric skin sample. Results again demonstrated a similar increase in both ADAM 10 and ADAM 17 protein levels in MCC tumour samples compared to control, which was MCPyV negative as indicated by the lack of ST and LT expression (Fig 3B and 3C). Moreover, we compared the MCPyV-negative MCC13 cell line versus two MCPyV-positive cells lines, WAGA and PeTa. Similar results were observed showing that the presence of MCPyV ST increases ADAM 10 and 17 protein levels (S1 Fig). Immunoblot analysis was also performed on cellular lysates of the MCPyV-positive MCC cell line, WAGA, transduced with lentiviruses containing a shRNA scrambled control or shRNA targeting ST, as previously described [31]. Results demonstrated that MCPyV ST depletion did not affect MCPyV LT levels but led to a reduction in ADAM 10 and ADAM 17 protein levels. Conversely, ST depletion leads to increased Alpha-E-catenin levels (Fig 3D). To confirm these observations and determine if ADAM 10 transcripts are significantly increased in MCPyV-positive MCC compared with MCPyV-negative MCC, gene expression profiles for a total of ninety-four patients were obtained from a publicly available dataset (accession number GSE39612 [9]). Bioinformatic analysis identified a significant increase (2.5 fold, p = 0.03) in ADAM 10 expression in MCPyV-positive MCC compared with MCPyV-negative MCC control samples. Moreover, a similar analysis was performed to analyse ADAM protein expression in control GFP versus MCPyV ST expressing cell datasets (accession number GSE79968) [26]. A significant increase in both ADAM 10 (p = <0.0001) and ADAM 17 (p = <0.0001) was observed upon 48 hours MCPyV ST expression. Together these data suggest that ADAM 10 and 17 protein levels are increased upon MCPyV ST expression and in MCPyV-positive MCC tumour samples.

### ADAM 10 and 17 localisation at the plasma membrane is increased upon MCPyV ST expression

**Fig 4:**
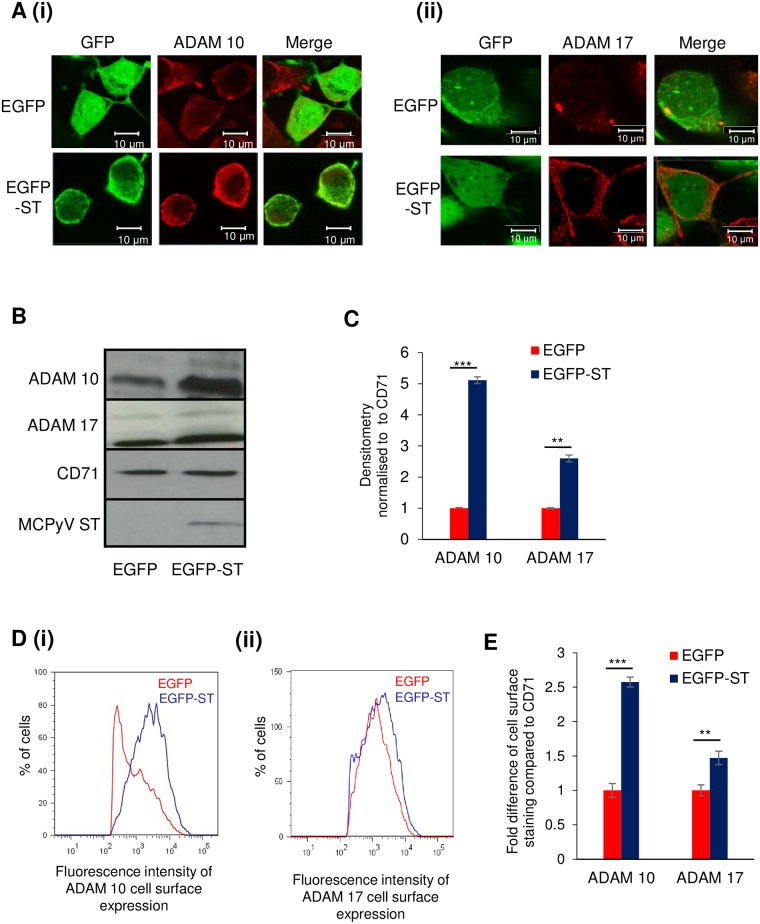
ADAM 10 and 17 localisation at the cell surface is increased upon MCPyV ST expression. (A) HEK 293 cells were transfected with 1 μg of EGFP or EGFP-ST expression plasmids. 24 hours later cells were fixed and GFP fluorescence analysed by direct visualisation, whereas endogenous (i) ADAM 10 and (ii) ADAM 17 were identified by indirect immunofluorescence using specific antibodies. (B) Surface biotinylation experiments were performed in EGFP or EGFP-ST transfected HEK 293 cells, lysates were probed for ADAM 10, ADAM 17 and CD71 as a surface marker control. (C) Densitometry of immunoblots was performed using ImageJ software. Data analysed using three replicates per experiment, n = 3 and statistical analysis using a two-tailed t-test with unequal variance, *** = p<0.001, ** = p<0.01. (D) EGFP or EGFP-ST transfected HEK 293 cells were harvested and stained with (i) ADAM 10-, (ii) ADAM 17- and CD71-specific antibodies. Mean fluorescence intensity was analyzed using FlowJo software. (E) Fold difference of cell surface staining was calculated using three replicates per experiment, n = 3 by a two-tailed t-test with unequal variance, *** = p<0.001 and ** = p<0.01.

For active ADAM proteins to cleave their chosen substrate, they are required to be present at the same subcellular location [59]. As adhesion molecule receptors are localised at the plasma membrane, we next determined whether MCPyV ST enhancement of ADAM 10 and 17 protein levels led to their accumulation at the plasma membrane [60]. HEK 293 cells transfected with EGFP or EGFP-ST were fixed and stained for endogenous ADAM 10 and ADAM 17 in non-permeabilised cells. MCPyV ST-expressing cells showed increased levels of both ADAM 10 and 17 proteins at the plasma membrane, in comparison to the EGFP control cells (Fig 4A). To confirm these results, cell surface accumulation of ADAM proteins was measured by surface biotinylation assays in EGFP versus EGFP-ST expressing HEK 293 cells. Immunoblotting of surface biotinylated proteins confirmed that MCPyV ST expression specifically increased the plasma membrane levels of ADAM 10 and 17 proteins, in contrast the control cell surface protein, CD71, showed no such increase (Fig 4B). Densitometry-based quantification of the immunoblot analysis showed a significant increase in both ADAM 10 and 17 accumulation at the plasma membrane by 5 fold and 2.5 fold, respectively (Fig 4C). Further validation was performed using flow cytometry with ADAM 10- and ADAM 17-specific antibodies (Fig 4D and 4E). Notably however, both assays showed a greater accumulation of ADAM 10 compared to ADAM 17 at the cell surface. Together, these results suggest that MCPyV ST expression results in the accumulation of cellular sheddases, primarily ADAM 10, at the plasma membrane.

### ADAM 10 is required for MCPyV ST-induced cell junction disruption

**Fig 5:**
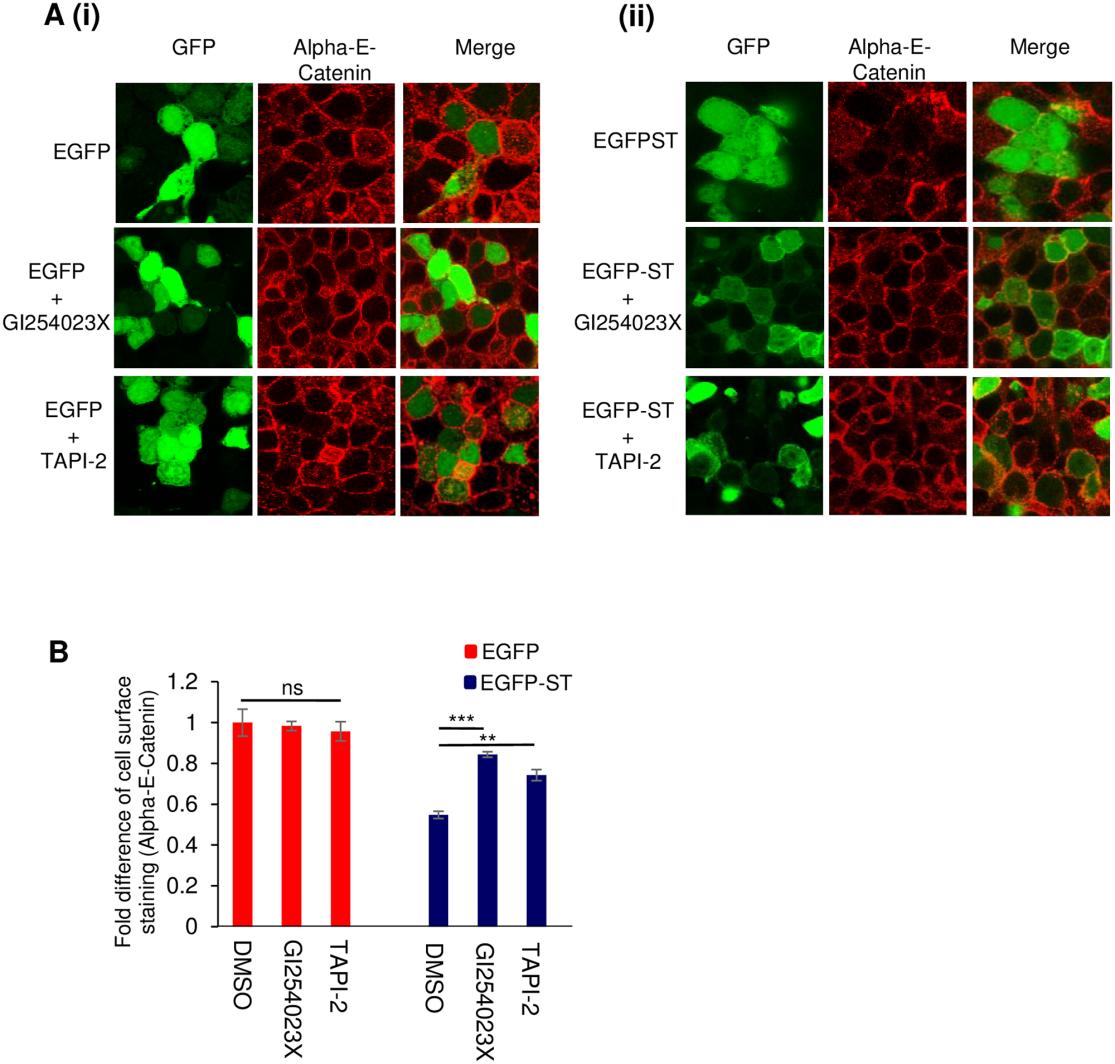
ADAM 10 is required for MCPyV ST-induced cell junction disruption. (A) HEK 293 cells were transfected with 1 μg of (i) EGFP or (ii) EGFP-ST expression plasmids and grown in the absence or presence of GI254023X or TAPI-2 inhibitors. 24 hours later cells were fixed and EGFP fluorescence analysed by direct visualisation, whereas endogenous Alpha-E-Catenin was identified by indirect immunofluorescence using a specific antibody. The top panel for both (i) and (ii) is the same as Fig 1A. (B) EGFP or EGFP-ST transfected HEK 293 cells were grown in the absence or presence of GI254023X or TAPI-2 inhibitors for 24 hours, then harvested and stained with an Alpha-E-catenin specific antibody and Alexa-Fluor-tagged secondary antibody. Mean fluorescence intensity was analyzed using FlowJo software. Fold difference of cell surface staining was calculated using three replicates per experiment, n = 3 by a two-tailed t-test with unequal variance, *** = p<0.001 and ** = p<0.01.

To determine whether ADAM protein accumulation at the plasma membrane is implicated in the observed disruption of cell junctions upon MCPyV ST expression, EGFP and EGFP-ST HEK 293-expressing cells were incubated in the absence or presence of two distinct ADAM protease inhibitors. MTS assays identified non-cytotoxic concentrations of an ADAM 10-specific inhibitor (GI254023X) and dual ADAM 10/17 inhibitor (TAPI-2) (S2 Fig), no specific ADAM 17 inhibitor is commercially available. Following a 24 hour incubation period, cells were fixed and non-permeabilised cells stained with an Alpha-E-catenin-specific antibody. As previously shown in Fig 1, incomplete staining of the cell junctions was observed in MCPyV ST-expressing cells, compared to control EGFP cells. However, retention of the cell junctions was observed in the presence of both the ADAM 10-specific and dual ADAM 10/17 inhibitors, implying that inhibition of ADAM sheddase activity, and specifically ADAM 10, is sufficient to prevent MCPyV ST-induced breakdown of cell-cell junctions (Fig 5A). Importantly, there was no observed change in the cell junction staining in EGFP control cells after incubation with either inhibitor. The inhibition of MCPyV ST-induced cell junction breakdown was also confirmed by quantifying the cell surface levels of Alpha-E-catenin using flow cytometry in EGFP versus EGFP-ST-expressing cells. Results demonstrated increased levels of Alpha-E-catenin expression at the cell surface upon addition of the inhibitors (Fig 5B). Notably, taking into consideration the greater accumulation of ADAM 10 over ADAM 17 at the plasma membrane in MCPyV ST-expressing cells and no enhancement of Alpha-E-catenin expression at cell junctions in the presence of the dual ADAM10/17 inhibitor over the ADAM 10 inhibitor alone, these results suggest that ADAM 10 may be the main cellular sheddase required for MCPyV ST-induced cell junction disruption.

### ADAM 10 is required for MCPyV ST-induced cell dissociation

**Fig 6:**
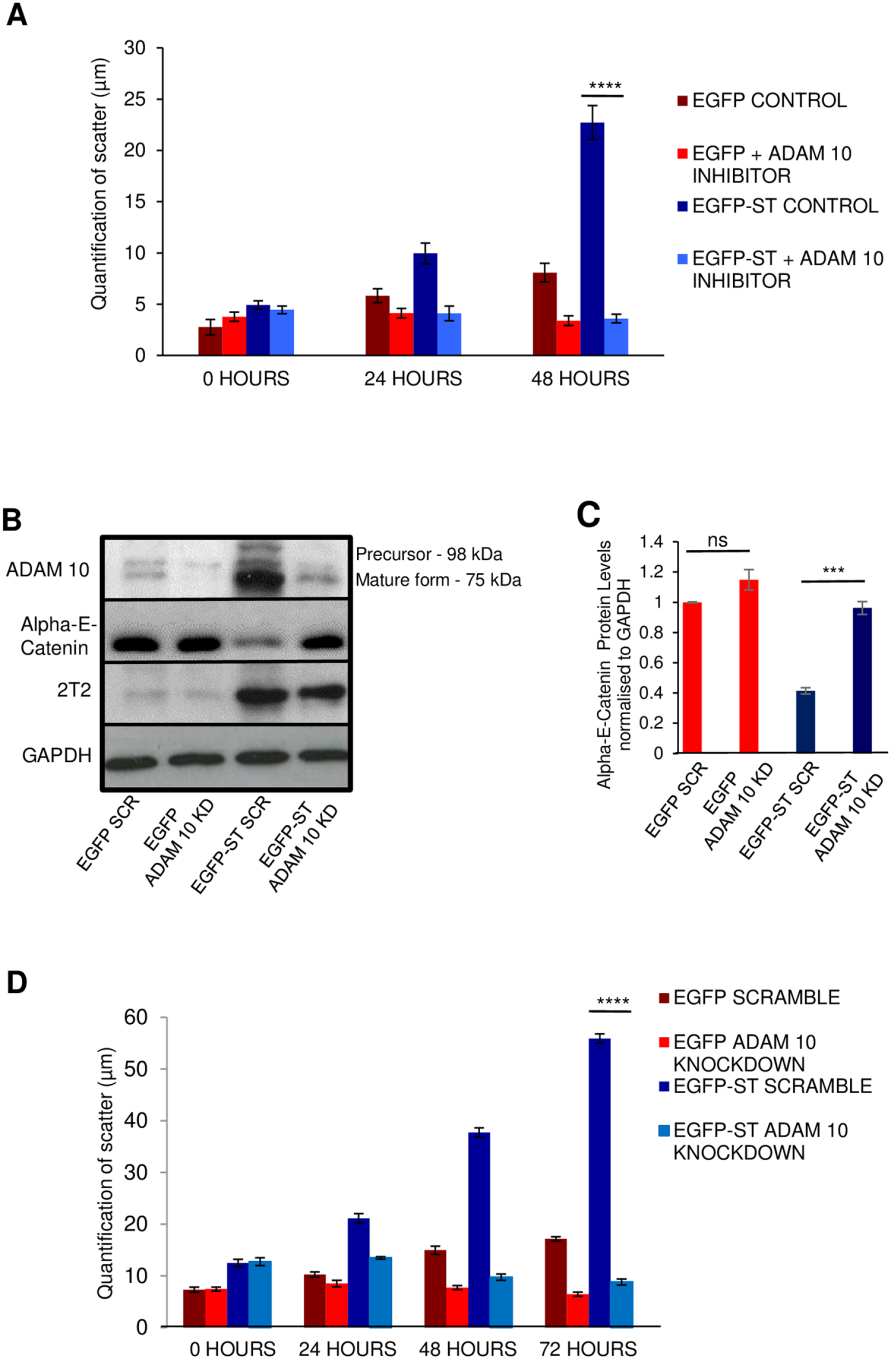
ADAM 10 is required for MCPyV ST-induced cell dissociation. (A) EGFP or EGFP-ST transfected HEK 293 cells were incubated with the ADAM 10 specific inhibitor, GI254023X (50 μM), then serum starved for 24 hours to induce aggregate formation. Upon reintroduction of serum, cells were fixed and stained with DAPI at 24 hourly intervals. Images were analysed using Image-J to quantify the distance between each cell nucleus. Data analysed using three replicates per experiment, n = 50 cells, by a two-tailed t-test with unequal variance, **** = p≤ 0.0001. (B) HEK 293 cells were transfected with 1 μg EGFP or EGFP-ST in the presence of either scramble or ADAM 10-specific siRNAs. After 24 hours, cell lysates were probed using ADAM 10- and Alpha-E-catenin specific antibodies. GAPDH was used to measure equal loading. 2T2 was used to probe for MCPyV ST expression. (C) Densitometry of immunoblots was performed using ImageJ software. Data analysed using three replicates per experiment, n = 3 and statistical analysis using a two-tailed t-test with unequal variance, *** = p<0.001. (D) HEK 293 cells were transfected with 1 μg EGFP or EGFP-ST in the presence of either scramble or ADAM 10-specific siRNAs, then serum starved for 24 hours to induce aggregate formation. Upon reintroduction of serum, cells were fixed and stained with DAPI at 6 hourly intervals. Images were analysed using Image-J to quantify the distance between each cell nucleus. Data analysed using three replicates per experiment, n = 50 cells, by a two-tailed t-test with unequal variance, **** = p≤ 0.0001.

To confirm that ADAM 10 was required for the enhanced cell dissociation observed in MCPyV ST-expressing cells, the cell scatter assay was repeated in EGFP control and MCPyV ST-expressing cells, in the absence and presence of the ADAM 10 specific inhibitor, GI254023X, at non-cytotoxic concentrations. Addition of GI254023X resulted in little change in the EGFP-expressing control cells. However, a significant decrease in cell dissociation, over the course of 48 hours, was observed in the presence of GI254023X compared to DMSO-treated MCPyV ST-expressing cells (Fig 6A). A similar level of cell dissociation inhibition was also observed using the ADAM10/17 dual inhibitor, TAPI-2 (S3 Fig), showing that no enhancement of inhibition is seen by targeting both ADAM 10 and 17. To confirm the specific role of ADAM 10 in MCPyV ST-induced cell dissociation, siRNA-mediated depletion of ADAM 10 was performed in EGFP and EGFP-ST-expressing HEK 293 cells (Fig 6B). Immunoblotting confirmed that MCPyV ST depletion led to Alpha-E-catenin protein levels comparable to EGFP control cells (Fig 6B and 6C). Cell scatter assays were then repeated in EGFP control or MCPyV ST-expressing cells, in the presence of either scrambled or ADAM 10-specific siRNAs. Depletion of ADAM 10 resulted in a similar reduction in cell dissociation levels observed with the specific ADAM 10 inhibitor (Fig 6D). These data therefore suggest that ADAM 10 is required for the increased ability of cells to dissociate upon MCPyV ST expression.

### ADAM 10 inhibition impedes the ability of MCPyV ST expressing cells to migrate

**Fig 7:**
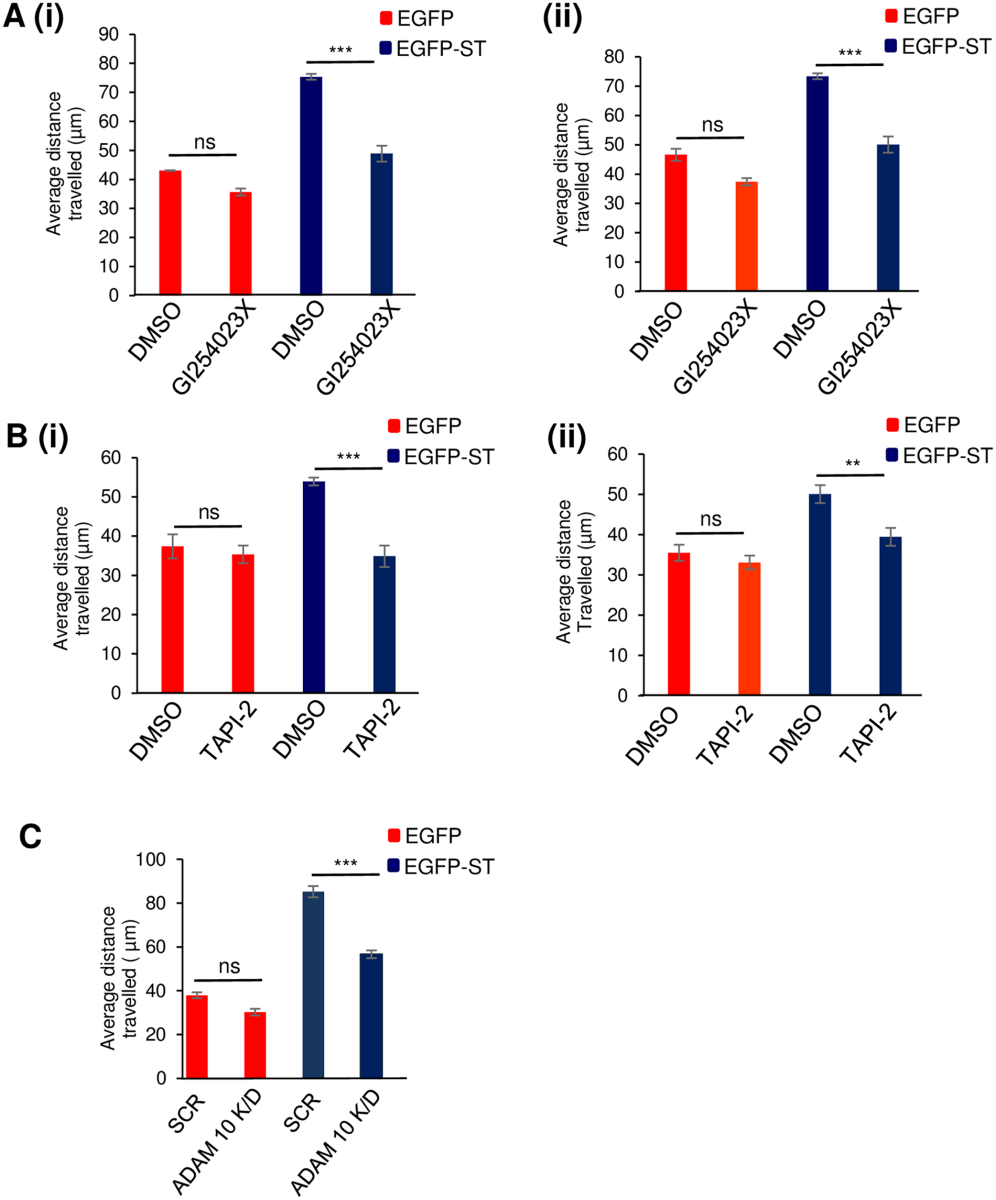
ADAM 10 inhibition impedes the ability of MCPyV ST expressing cells to migrate. (A) EGFP or EGFP-ST transfected (i) HEK 293 or (ii) MCC13 cells were incubated with DMSO or the ADAM 10 specific inhibitor, GI254023X (50 μM). (B) EGFP or EGFP-ST transfected (i) HEK 293 or (ii) MCC13 cells were incubated with DMSO or the ADAM 10/17 dual inhibitor, TAPI-2 (50 μM). (C). HEK 293 cells were transfected with 1 μg EGFP or EGFP-ST in the presence of either scramble or ADAM 10-specific siRNAs. After 24 hours, cell motility was analysed using an IncuCyte Zoom-kinetic live cell imaging system. Images were taken every 30 minutes for a 24 hour period. The movement of cells were then tracked using Image J software and the average distance travelled was measured in μm (n = 50 per condition) and significance was tested using a 3-tailed Student’s t-test, *** = p<0.001 and ** = p<0.01.

ADAM-mediated shedding of cell adhesion molecules may also stimulate cell signalling pathways to induce cell motility [30, 31]. Therefore, we next examined if ADAM proteins have any downstream impact on the motility and migratory potential of MCPyV ST-expressing cells. Here, the migrating potential of EGFP control and EGFP-ST HEK 293 and MCC13-expressing cells were assessed using Incucyte kinetic live cell imaging, in the absence or presence of non-cytotoxic concentrations of the ADAM 10-specific (GI254023X) and dual ADAM 10/17 (TAPI-2) inhibitors. Incubation of the ADAM 10 (GI254023X) inhibitor showed a slight but insignificant decrease in the motility of EGFP control cells, implying that any changes observed in migratory rates of MCPyV ST expression cells is not due to changes in cell viability or cytotoxicity. In contrast, ADAM 10 inhibition resulted in a significant decrease in the distance travelled of MCPyV ST-expressing cells, reminiscent of control cell migration (Fig 7A). A similar trend was also observed with the dual ADAM 10/17 (TAPI-2) inhibitor (Fig 7B), suggesting that inhibition of ADAM 10 alone was sufficient to repress the MCPyV ST-induced cell migratory phenotype. To validate the use of ADAM-specific inhibitors, similar live cell imaging motility assays were also performed in ADAM 10-depleted EGFP and MCPyV ST-expressing HEK 293 cells, which resulted in a reduction in the motility of MCPyV ST-expressing cells, to levels similar to control EGFP-expressing cells (Fig 7C).

**Fig 8:**
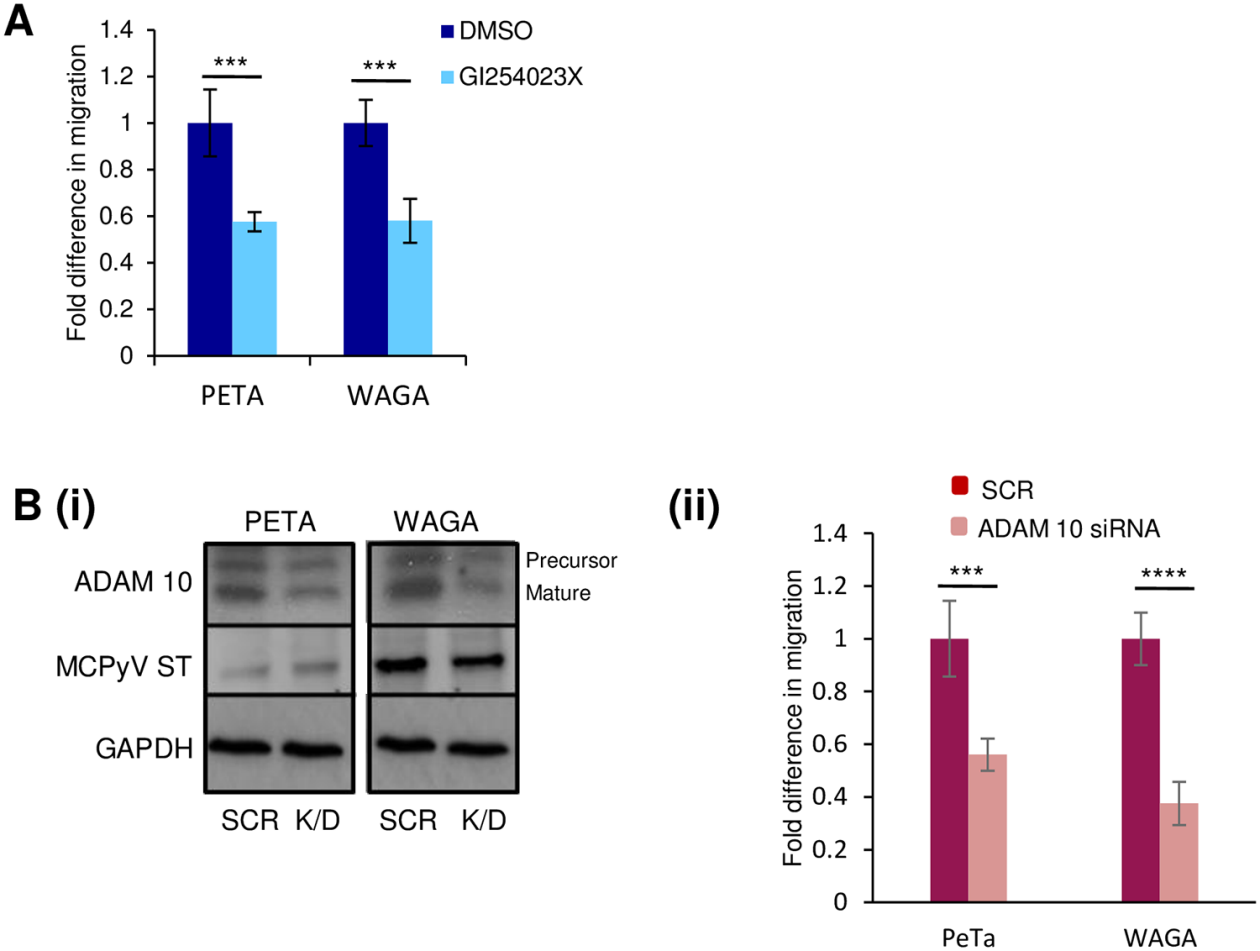
ADAM 10 is required for the motility of MCC cells. (A) MCPyV positive MCC cell lines, PeTa and WAGA, were incubated with DMSO or the ADAM 10 specific inhibitor, GI254023X (50 μM). Cells were then transferred into migration wells and allowed to migrate from serum-free to 10% FBS conditions for 24 hours. Migratory cells were stained and measured at 560 nm to quantify migration. Average cell migration was calculated and significance tested using a two-tailed Student’s t-test (n = 3), *** = p<0.001. (B) (i) PeTa and WAGA cells were transfected with either scramble or ADAM 10-specific siRNAs and cell lysates probed to confirm successful knockdown with an ADAM 10-specific antibody. GAPDH was used as loading control. 2T2 was used to probe for MCPyV ST expression. (ii) Control and ADAM 10-depleted cells were then transferred into migration wells and allowed to migrate from serum-free to 10% FBS conditions for 24 hours. Migratory cells were stained and measured at 560 nm to quantify migration. Average cell migration was calculated and significance tested using a two-tailed Student’s t-test (n = 3), **** = p<0.0001, *** = p<0.001.

To demonstrate that ADAM 10 is required for cell motility and migration of MCPyV-positive MCC cell lines, haptotaxis migration assays were performed. This assay investigates the three-dimensional migration of cells towards a chemoattractant across a permeable chamber. Two MCPyV-positive MCC cell lines, WAGA and PeTa, were incubated in the absence or presence of the ADAM 10 inhibitor (GI254023X) at non-toxic concentrations assessed by MTS assay (S4 Fig) or upon siRNA-mediated scramble or ADAM 10-specific depletion. After treatment, cells were allowed to migrate for 24 h before migration was assessed by immunofluorescent staining of cells that had migrated into the chambers. Results showed that migration of MCPyV positive MCC cell lines were significantly reduced compared to control, upon treatment with GI254023X (Fig 8A) or upon ADAM 10 depletion (Fig 8B), suggesting that MCPyV positive MCC cell line migration is ADAM 10 dependent. Together, these results suggest that ADAM 10 is required for MCPyV ST-mediated enhanced cell motility and migration.

## Discussion

MCPyV ST has emerged as the major transforming factor in MCPyV-positive MCC. Recently we reported a potential role for MCPyV ST in MCC metastasis, whereby ST cultivates a pro-migratory cell phenotype by destabilising microtubules [30], inducing filopodia formation [31] and modulating cellular chloride channels [32]. Cancer metastasis occurs via a series of complex events that are collectively known as the invasion-metastasis cascade [61]. The apex event in the metastatic cascade is broadly accepted to be mediated by an EMT, providing tumour cells increased motility allowing invasion of the ECM. Most oncoviruses have been shown to manipulate the EMT axis, for example, human papillomavirus 16, Epstein-Barr virus (EBV), hepatitis B virus and the polyomavirus simian virus 40 have all been shown to induce metastasis, through a variety of mechanisms including; cellular adhesion complexes, cytoskeletal reorganisation and gene expression modulation [62–65]. EBV latent membrane protein-1, for example orchestrates EMT via several different routes, including the transcriptional repression of E-cadherin via activation of DNA methyltransferases [66] and increased expression of the pleiotropic EMT transcription factors, Twist and Snail [67, 68].

Here we expand on recent observations suggesting that MCPyV ST can trigger elements of the EMT and initiate the invasion-metastasis cascade, by demonstrating that MCPyV ST induces cell-surface expression of cellular sheddases, specifically ADAM 10 and 17. Moreover, we show that MCPyV ST-mediated induction of ADAM 10 is required for MCPyV ST-induced cell-cell junction disruption which in turn enhances cell dissociation, migration and invasion. Although we focus herein on the link between MCPyV ST induction of ADAM proteins in metastatic spread, it must be noted that activation of ADAM10 may also serve in MCPyV fitness. Fibroblasts are a target of MCPyV infection [69] and is it known that MCPyV is shed from the surface of the skin, it is plausible therefore ADAM10 expression be a way for infected fibroblasts to migrate into the epidermis or hair follicle so the virus can be shed into the environment. How MCPyV ST regulates ADAM 10 expression is not yet clear, although results suggest this is likely to be at the transcriptional level. The ADAM 10 promoter contains functional binding sites for Sp1 and USF [70] and has been reported to be activated by numerous transcriptional activators including, XBP1, JUN, ACAD8, PPARG, SCAND1 and ITGB3BP [71, 72]. Interestingly, ACAD8, PPARG and ITGB3BP all appear in a recent RNA-seq data set of MCPyV ST-induced genes [26], raising the possibility that these transcription factors may be responsible for MCPyV ST-mediated induction of ADAM 10 expression.

There is a growing appreciation for the role played by ADAM proteins in numerous human diseases [73], including Alzheimer’s disease, cardiovascular disease, rheumatoid arthritis and cancer [52]. The best characterised sheddase in terms of cancer aetiology is ADAM 17, which is implicated in the development and progression of numerous neoplasms [74]. ADAM 17 came to prominence due to its ability to shed the soluble form of the inflammatory cytokine, TNFα from it precursor product [75, 76], however, despite TNFα being widely implicated in tumour development and progression, it is the ability of ADAM 17 to hydrolyse and promote the release of epidermal growth factor receptor (EGFR)/human EGFR (HER) precursor ligands that features most frequently in published studies. For example, ADAM 17-mediated shedding of TGFβ is implicated in breast [77, 78] and renal [79] cancer progression. Moreover, release of the transmembrane protein with EGF and two follistatin motifs (TMEFF2) increases prostate cancer cell motility [80]. We observed significant upregulation of ADAM 17 in response to MCPyV ST expression and in MCC tumours, however, comparison of ADAM 10 and ADAM 10/17 inhibitor experiments suggest that ADAM 17 is not required for the EMT-associated phenotypes observed following expression of MCPyV ST. This supposition is supported by bioinformatic analysis of MCPyV-positive MCC compared with MCPyV-negative MCC tumours, which identified significantly increased expression of ADAM 10, but not ADAM 17 in 94 patient samples. The role of ADAM 10 in cancer metastasis is less clear, however emerging evidence suggests that ADAM 10 maybe cell-type specific, driving motility and invasion in breast [81], pancreatic [82], melanoma [83] and bladder [84] metastasis compared with primary tumours, but having alternative effects on proliferation in other tissue types. Interestingly, while HER ligand release is generally ADAM-specific, overexpression of individual ADAM proteins drives promiscuity in terms of ligand cleavage [85]. This raises the possibility that MCPyV ST-induced overexpression may enable ADAM 10 to cleave proteins ordinarily regulated by other sheddases, a scenario that needs to be considered when investigating downstream targets of ADAM 10 in MCC.

Generally, metastasised MCC is treated with various regimens of broad-spectrum chemotherapy agents. However, metastatic MCC responses are not robust and often associated with high toxicity in elderly patients [86]. Response rates range from 52% to 61% in the distant metastatic setting, with progression-free survival (PFS) and overall survival typically measured in months [87–89]. One of the strongest predictors for survival is a high level of intratumoural CD8+ T cells most frequently observed in MCPyV-positive MCC [90, 91]. MCPyV-specific CD8+ T cells express high levels of PD-1 and TIM-3 (the T cell immunoglobulin and mucin domain-3), which prompted immunotherapy-based clinical trials in MCC patients with the anti-PD-1 antibodies, pembrolizumab [92] and avelumab [93]. Both phase 2 trials reported encouraging and positive response rates with improved PFS, leading to pembrolizumab being listed as a treatment option for late-stage MCC in the National Comprehensive Cancer Network 2017 guidelines and avelumab being granted accelerated FDA approval as a first-line treatment for metastatic MCC. Whilst promising, around half of the patients involved in these clinical trials derived limited benefit from either drug [94], indicating the importance of identifying additional agents to use in combination with anti-PD-1 antibodies. This approach may have exciting possibilities for ADAM 10/17 inhibitors, as TIM-3 is shed by both ADAM 10 and 17 and ADAM 10 cleaves MHC-I [95]. Notably, monoclonal antibody blocking of TIM-3 reduced PD-1 expression and increased cytokine production [96], indicating that TIM-3 functions to dampen the immune system [97]. Therefore, ADAM 10 and 17 inhibitors may stimulate the immune system by reducing TIM-3 cleavage.

One of the most widely characterised ADAM inhibitory compounds is INCB3619 (Incyte), a dual ADAM 10 and 17 inhibitor which inhibits the catalytic activity of ADAM proteins by chelating zinc at the active site [53]. *In vitro* studies using breast and small cell lung cancer cell lines, have shown that INCB3619 reduced the cleavage of HER2 and amphiregulin, thereby sensitising cells to the EGFR tyrosine kinase inhibitor, gefintinib or a dual EGFR/HER2 inhibitor, GW2974 [98–100]. These observations have also been extended in animal models where INCB3619 shows anti-cancer activity against malignancies of the lung (non-small cell), breast, head and neck [98, 99]. Notably, a structurally similar compound with enhanced pharmokinetic properties, IMCB7839 (Aderbasib), has undergone phase I/II clinical trials in patients with HER2-positive breast cancer, in combination with Herceptin (trastuzumab). Results showed improved clinical responses in a subset of HER2-positive metastatic breast cancer patients, expressing the p95 form of HER2 [52, 98]. At present, additional phase I/II clinical trials are ongoing, for example in patients with diffuse large B cell non-Hodgkin lymphoma using INCB7839 in combination with the monoclonal antibody rituximab [52]. Therefore, given our data showing a significant upregulation of ADAM 10/17 in MCC cell lines and tumours and the integral role played by ADAM 10 in MCPyV ST-mediated enhanced cell dissociation and invasion, selective inhibitors of ADAM 10 and 17 may prove to be potent novel therapeutics when given in combination with immune checkpoint inhibitors for the treatment of advanced MCC.

## Materials and methods

### Plasmids, siRNAs, antibodies and chemicals

The expression vectors for EGFP-ST has been previously described [23, 30, 31]. MCPyV ST-tagging shRNA plasmids were kindly provided by Dr Masa Shuda, Pittsburgh. ADAM 10 and 17-specific siRNAs were purchased from Dharmacon. Antibodies against ADAM 10, ADAM 17, ADAM TS1, and GAPDH were purchased form Abcam and used at a dilution range of 1:100–1:500, the ZO-1, CD71 and Alpha-E-catenin antibodies were purchased from Cell signalling and used at 1:100 dilution. The 2T2 hybridoma was provided by Dr Buck, National Cancer Institute, Bethesda, MD. All antibodies used for immunofluorescence were diluted 1:200. ADAM 10 specific inhibitor, GI254023X and ADAM 10/17 dual inhibitor, TAPI-2 where purchased from TOCRIS and Merck Millipore, respectively. Cell toxicity was measured using a MTS-based CellTiter 96 AqueousOne Solution Proliferation assay (Promega), as previously described [101].

### Mammalian cell culture

HEK-293 Flip-In cell line was purchased from Invitrogen. i293-ST, i293-GFP, and i293-GFP-ST cell lines were derived from HEK-293 Flip-Ins using manufacturer’s protocol as previously described [23]. HEK-293 cells were obtained from ECACC and were maintained in Dulbecco’s modified Eagle’s medium (DMEM) containing 10% foetal bovine serum (FBS) and 1% penicillin/streptomycin as previously described [102]. The MCPyV negative cell line MCC13 (ECACC) and positive MCC cell lines, WAGA and PeTa (ATCC), were grown in RPMI 1640 (Sigma) supplemented with 10% FBS. ST-FLAG, EGFP and EGFP-ST expression was induced from i293-ST, i293-GFP, and i293-GFP-ST cells respectively with 2 μg/ml Doxycycline hyclate for up to 48 hours. Cells were plated into 6-well plates and transfections routinely used 1 μg plasmid DNA and Lipofectamine 2000 (Life Technologies) or 5 μg plasmid DNA and nucleofection (Lonza) following the manufacturer’s instructions.

### Immunofluorescence

Immunofluorescence was carried out as previously described [103]. If appropriate, cells were treated with inhibitors for24 hours prior to fixation. Cells were viewed on a Zeiss LSM880 confocal laser scanning microscope under an oil-immersion 63x objective lens. Images were analysed using the LSM imaging software as previously described [104].

### Flow cytometric detection of cell-surface molecules

EGFP and EGFP-ST-transfected cells were detached using Versene (Sigma-Aldrich). The harvested cells were washed with ice-cold PBS and resuspended at 2x10^6^ cells/ml in freshly made staining buffer (PBS, 10% FCS, 3% BSA). Cells were then incubated with appropriate dilutions of primary antibody or staining buffer for 1 hour at room temperature in the dark, washed with staining buffer and then incubated with Alexa-Fluor-tagged secondary antibodies or staining buffer for 1 hour at room temperature. Cells were washed twice in PBS with centrifugation (350x g, 5 min) and then analyzed by flow cytometry on a FACSCalibur, (BD Bioscience, Wokingham, UK) and the data analyzed using FlowJo software (Tree Star, Ashland, OR, USA).

### Immunoblotting

Skin and MCC tumour biopsy samples were crushed using a pestle and mortar on dry ice, and homogenised by sonication prior to lysis in RIPA buffer (50 mM Tris-HCl pH 7.6, 150 mM NaCl, 1% NP40), supplemented with protease inhibitor cocktail (Roche) as previously described [105]. Proteins were separated by SDS-PAGE, transferred to nitrocellulose membranes and probed with the appropriate primary and HRP-conjugated secondary antibodies. Proteins were detected using EZ-ECL enhancer solution (Geneflow) as previously described [106]. Densitometry was performed using ImageJ software.

### qRT-PCR

RNA was extracted using TRIzol (Invitrogen) and DNase treated using the Ambion DNase-free kit, as per the manufacturer’s instructions, before RNA (1μg) from each fraction was reverse transcribed with SuperScript II (Invitrogen), as per the manufacturer’s instructions, using oligo(dT) primers (Promega). 10ng of cDNA was used as template in SensiMixPlus SYBR qPCR reactions (Quantace), as per manufacturer’s instructions, using a Rotor-Gene Q 5plex HRM Platform (Qiagen), with a standard 3-step melt program (95 °C for 15 seconds, 60 °C for 30 seconds, 72 °C for 20 seconds) as previously described [107]. With GAPDH as internal control mRNA, quantitative analysis was performed using the comparative ΔΔCt method as previously described [108].

### Cell scatter assay

EGFP and EGFP-ST-transfected HEK 293 cells were seeded in DMEM containing 10% FBS at a density of 2 × 10^4^ per 35 mm culture dish. 18 hours later, cells were serum starved for 24 hours to induce aggregate formation. Upon reintroduction of serum, cells were fixed and stained with DAPI at 6 hourly intervals and clusters of cells were imaged using a Zeiss LSM880 confocal laser scanning microscope using a 10x objective lens. Images were analysed using the LSM imaging software to quantify the distance between each cell nucleus.

### Multicolour immunohistochemistry

Formalin-fixed, paraffin-embedded (FFPE) sections from primary MCC tumours were purchased from Origene and analysed as previously described [32]. Primary antibodies were: FITC-conjugated anti-CK20 (Dako, dilution 1:50), MCPyV LT CM2B4 (Santa Cruz Biotechnology, dilution 1:125) and ADAM 10 and 17 (Abcam, dilution 1:250). An isotype-matched irrelevant antibody was used as a negative control on sections of tissues in parallel, a rabbit polyclonal isotype control antibody (Abcam) was used to match the ADAM 10 primary antibody. Sections were incubated with appropriate secondary antibodies labelled with different fluorochromes (Alexa Fluor 546 IgG2B, 643 IgG2A, Invitrogen, and IgG (H+L)-TRITC, Jackson ImmunoResearch). All slides were mounted with Immuno-Mount and images were captured with a Zeiss LSM880 confocal laser scanning microscope.

### Gene expression analysis

Metadata and pre-processed data (FPKM) were downloaded from Gene Expression Omnibus (GSE79968) [26] and GSE39612 [9]. Data were normalised by the trimmed mean of M-values methods using edgeR package to account for batch effects and differences in sequencing depth among the samples using R/Bioconductor [109]. The differential expression analysis was performed using the R Bioconductor packages, voom and limma.

### Cell surface biotinylation assay

Cell surface biotinylation was performed using the Pierce Cell Surface Protein Isolation kit (Thermo Scientific) according to the manufacturer’s protocol. Cells were incubated a cell-impermeable, cleavable biotinylation reagent, EZ-LINK Sulfo-NHS-SS-Biotin, to label exposed primary amines of proteins on the cell surface. After cell lysis, biotinylated cell surface proteins were affinity-purified using NeutrAvidin Agarose Resin (Thermo Scientific). Precipitated proteins were then analysed using immunoblotting with ADAM 10- and ADAM 17- specific antibodies. A CD71-specific antibody was used as a suitable loading control.

### Live cell imaging

Cell motility was analysed using an Incucyte kinetic live cell imaging system as directed by the manufacturer. HEK293 cells or i293-GFP/i293-GFP-ST cells were seeded at a density of 25,000 cells per well of a 6 well plate, MCC13 cells were seeded at a density of 100,000 cells per well of a 6 well plate. After 12 hours, the cells were transfected with 1 μg of DNA per well and/or induced using doxycycline hyclate. For transfected cells, media was changed after 6 hours (HEK-293 or derivatives) or 12 hours (MCC13). If appropriate, cells were treated with inhibitors for 24h pre-imaging. Imaging was performed for a 24 hour period, with images taken every 30 minutes. Cell motility was then tracked and analysed using ImageJ software.

### Haptotaxis migration assay

Migration assays were performed using a CytoSelect 24-well Haptotaxis Assay Collagen coated plates (Cell Biolabs, Inc), as directed by the manufacturer. All conditions were performed in triplicate.

## Supporting information

S1 Fig MCPyV ST expression induces cell dissociation in HEK 293 and MCC13 cells. (A) (i) i293-ST cells remained uninduced or were incubated for 24 h in the presence of doxycycline hyclate. Cells were then fixed and endogenous Alpha-E-catenin was identified by indirect immunofluorescence using a specific antibody. (ii) Western blotting using a FLAG and Alpha-E-catenin-specific antibodies confirm the expression of MCPyV ST in the induced i293-ST sample and also demonstrate reduced Alpha-E-catenin levels. (B) EGFP or EGFP-ST transfected MCC13 cells were serum starved for 24 hours to induce aggregate formation. Upon reintroduction of serum, cells were fixed and stained with DAPI at 6 hourly intervals. Images were analysed using Image-J to quantify the distance between each cell nucleus. Data analysed using three replicates per experiment, n = 50 cells, by a two-tailed t-test with unequal variance, *** = p≤ 0.001. (C) Summary of quantitative proteomic analysis previously published [30] showing an increase in ADAM proteins and a decrease in cell junction associated protein levels upon MCPyV ST expression. (D) Immunoblotting of MCPyV-negative MCC13 cells versus MCPyV positive MCC cell lines, PeTa and WAGA, using ADAM 10- and ADAM 17-specific antibodies. GAPDH was used as a measure of equal loading, the 2T2 hybridoma was used to confirm MCPyV ST expression.
(TIF)

S2 Fig Cell viability (MTS) assay for ADAM protein inhibitors. HEK 293 (A) and MCC13 (B) cells were treated with increasing concentrations of (i) ADAM 10 specific inhibitor, GI254023X or (ii) ADAM 10/17 dual inhibitor, TAPI-2 for 24 hours. 20 μl of the MTS reagent was added for 45 minutes and cell viability was measured at 492 nm using a plate reader.
(TIF)

S3 Fig An ADAM 10/17 dual inhibitor inhibits MCPyV ST-induced cell dissociation. EGFP or EGFP-ST transfected HEK 293 cells were incubated with the ADAM 10 and17 dual inhibitor, TAPI-2 (50 μM), then serum starved for 24 hours to induce aggregate formation. Upon reintroduction of serum, cells were fixed and stained with DAPI at 24 hourly intervals. Images were analysed using Image-J to quantify the distance between each cell nucleus. Data analysed using three replicates per experiment, n = 50 cells, by a two-tailed t-test with unequal variance, **** = p≤ 0.0001.
(TIF)

S4 Fig Cell viability (MTS) assay for ADAM 10 inhibitor in MCC cell lines. The MCPyV positive MCC cell lines PeTa (A) and WAGA (B) cells were treated with increasing concentrations of the ADAM 10 specific inhibitor, GI254023X. 20 μl of the MTS reagent was added for 45 minutes and cell viability was measured at 492 nm using a plate reader.
(TIF)
