# Supplementary figures and images for: Cellular sheddases are induced by Merkel cell polyomavirus small tumour antigen to mediate cell dissociation and invasiveness
Source: PLoS Pathog. 2018 Sep 6;14(9):e1007276. doi: 10.1371/journal.ppat.1007276 (PMC6143273; doi:10.1371/journal.ppat.1007276)

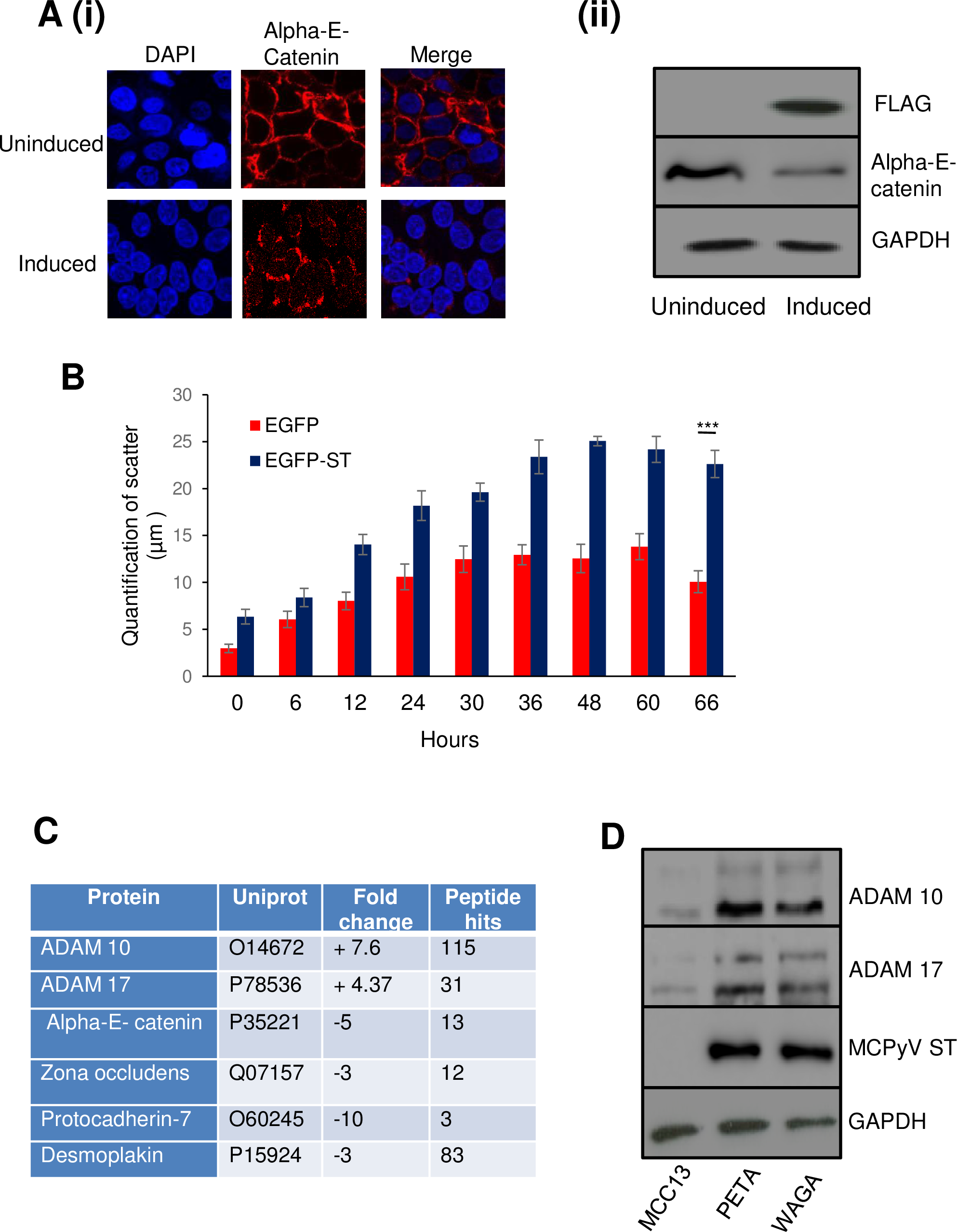

Supplement: S1 Fig — (A) (i) i293-ST cells remained uninduced or were incubated for 24 h in the presence of doxycycline hyclate. Cells were then fixed and endogenous Alpha-E-catenin was identified by indirect immunofluorescence using a specific antibody. (ii) Western blotting using a FLAG and Alpha-E-catenin-specific antibodies confirm the expression of MCPyV ST in the induced i293-ST sample and also demonstrate reduced Alpha-E-catenin levels. (B) EGFP or EGFP-ST transfected MCC13 cells were serum starved for 24 hours to induce aggregate formation. Upon reintroduction of serum, cells were fixed and stained with DAPI at 6 hourly intervals. Images were analysed using Image-J to quantify the distance between each cell nucleus. Data analysed using three replicates per experiment, n = 50 cells, by a two-tailed t-test with unequal variance, *** = p≤ 0.001. (C) Summary of quantitative proteomic analysis previously published [30] showing an increase in ADAM proteins and a decrease in cell junction associated protein levels upon MCPyV ST expression. (D) Immunoblotting of MCPyV-negative MCC13 cells versus MCPyV positive MCC cell lines, PeTa and WAGA, using ADAM 10- and ADAM 17-specific antibodies. GAPDH was used as a measure of equal loading, the 2T2 hybridoma was used to confirm MCPyV ST expression. (TIF) [file ppat.1007276.s001.tif]

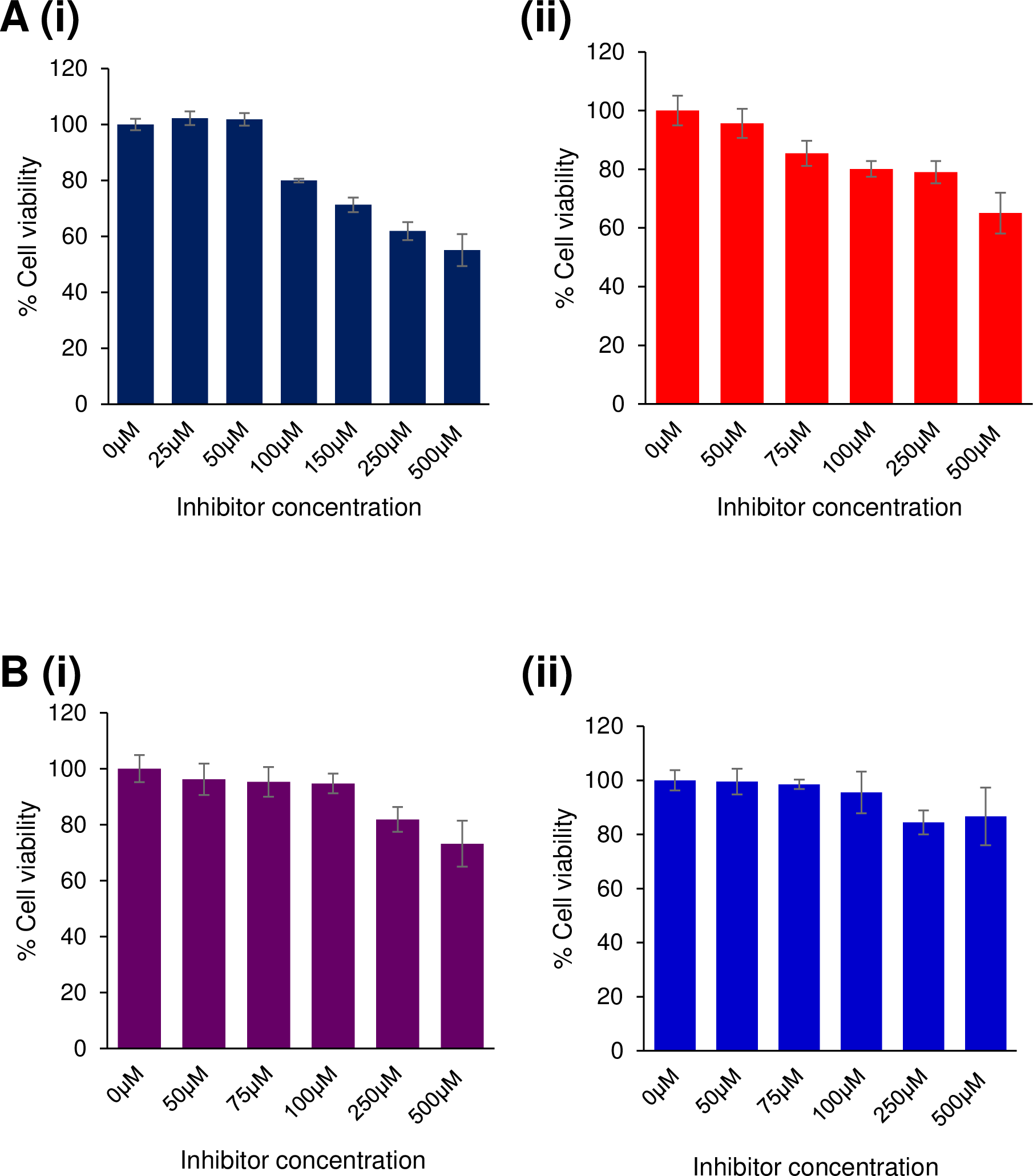

Supplement: S2 Fig — HEK 293 (A) and MCC13 (B) cells were treated with increasing concentrations of (i) ADAM 10 specific inhibitor, GI254023X or (ii) ADAM 10/17 dual inhibitor, TAPI-2 for 24 hours. 20 μl of the MTS reagent was added for 45 minutes and cell viability was measured at 492 nm using a plate reader. (TIF) [file ppat.1007276.s002.tif]

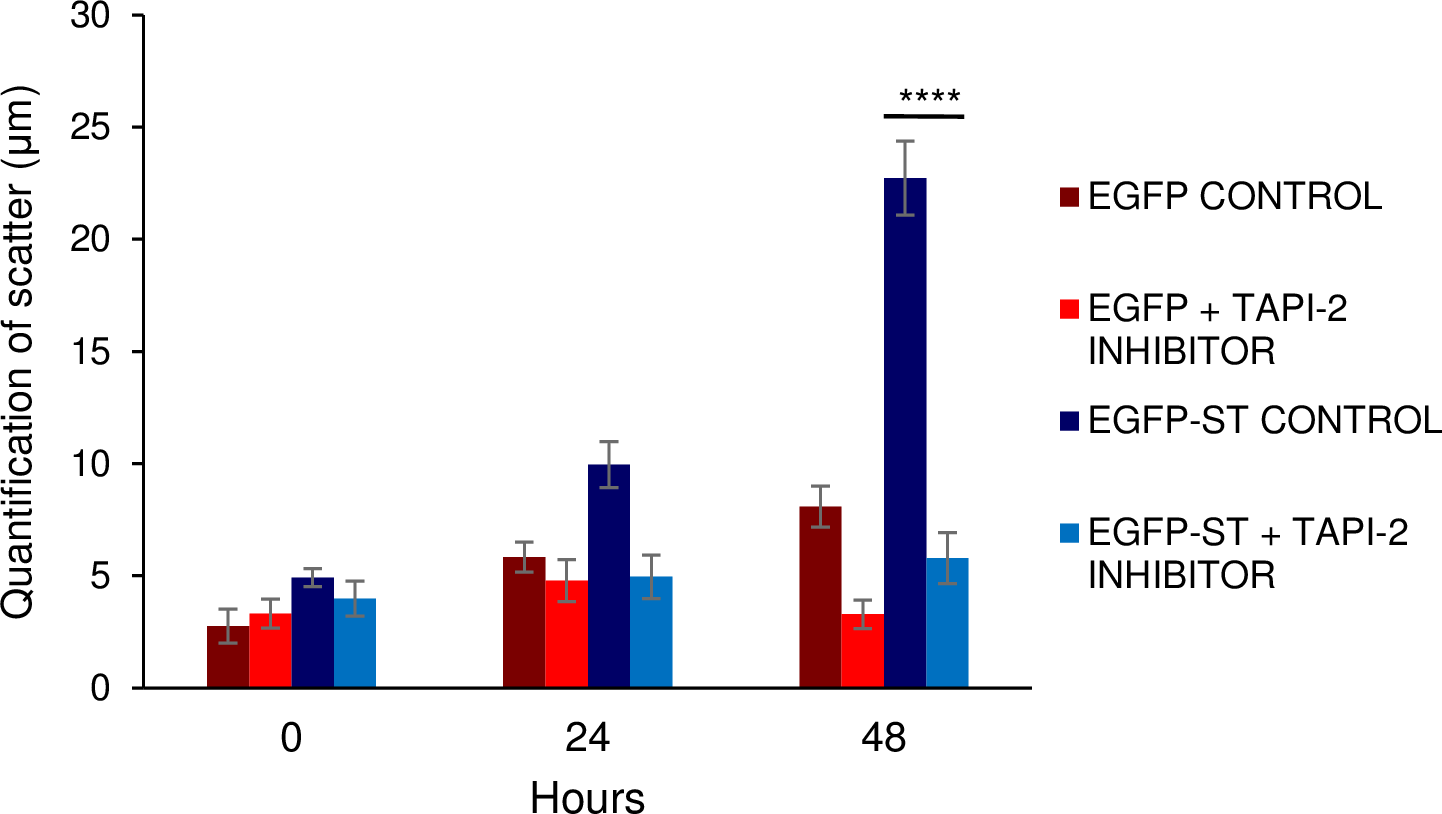

Supplement: S3 Fig — EGFP or EGFP-ST transfected HEK 293 cells were incubated with the ADAM 10 and17 dual inhibitor, TAPI-2 (50 μM), then serum starved for 24 hours to induce aggregate formation. Upon reintroduction of serum, cells were fixed and stained with DAPI at 24 hourly intervals. Images were analysed using Image-J to quantify the distance between each cell nucleus. Data analysed using three replicates per experiment, n = 50 cells, by a two-tailed t-test with unequal variance, **** = p≤ 0.0001. (TIF) [file ppat.1007276.s003.tif]

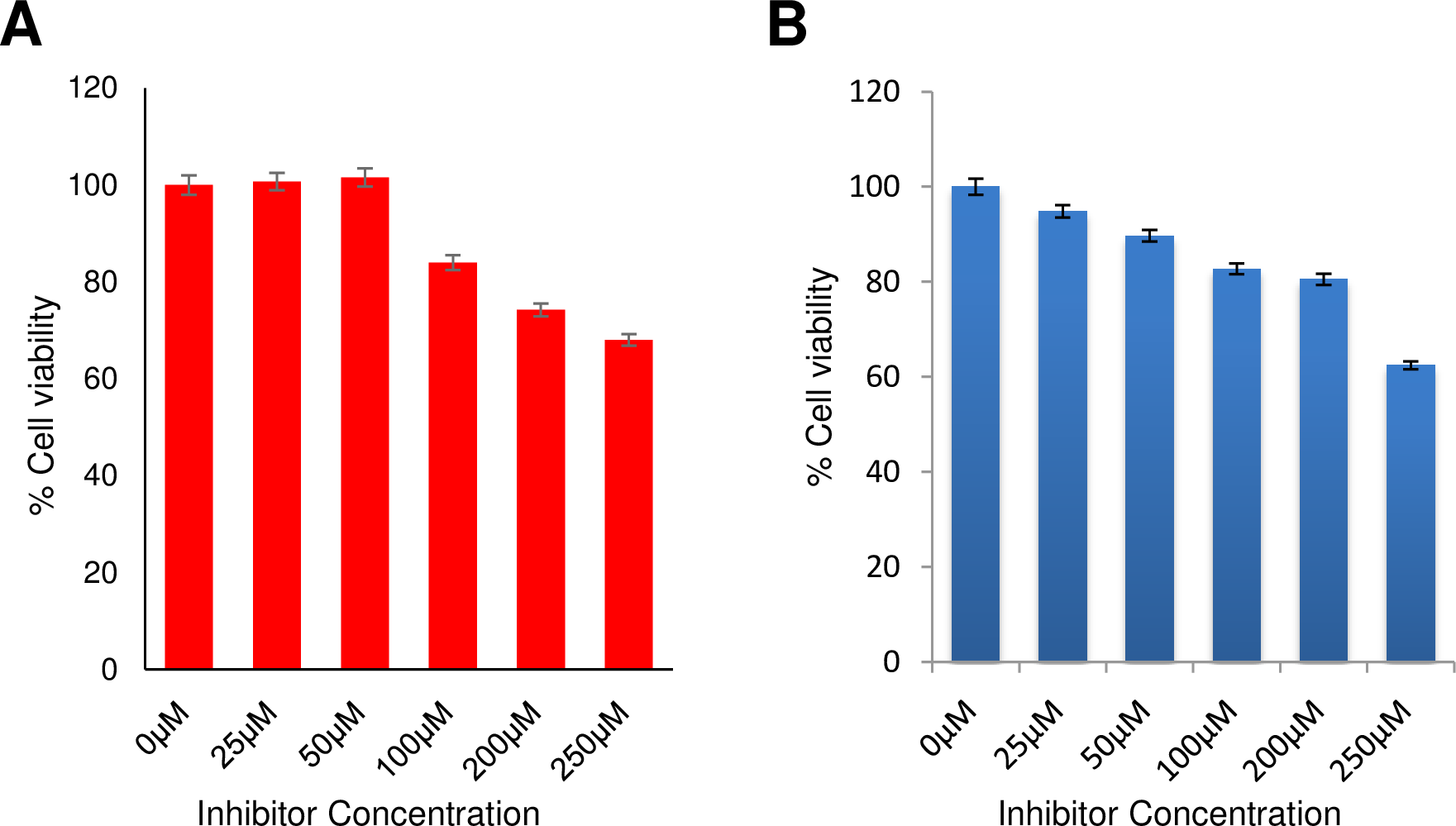

Supplement: S4 Fig — The MCPyV positive MCC cell lines PeTa (A) and WAGA (B) cells were treated with increasing concentrations of the ADAM 10 specific inhibitor, GI254023X. 20 μl of the MTS reagent was added for 45 minutes and cell viability was measured at 492 nm using a plate reader. (TIF) [file ppat.1007276.s004.tif]
